# Determinants of self-monitoring adherence in type 2 diabetes: bridging the gap in patient care

**DOI:** 10.3389/fpubh.2026.1784888

**Published:** 2026-04-01

**Authors:** Dana Hyassat, Kawther Alonezat, Maysoon Abdalrahim, Anwar Batieha, Yousef Khader, Sara Alnsour, Sarah B. Al-Qalalweh, Kamel Ajlouni

**Affiliations:** 1 National Center for Diabetes, Endocrinology and Genetics, The University of Jordan, Amman, Jordan; 2 School of Nursing, The University of Jordan, Amman, Jordan; 3 Department of Epidemiology and Biostatistics, Jordan University of Science and Technology, Irbid, Jordan; 4 School of Medicine, The University of Jordan, Amman, Jordan

**Keywords:** adherence to SMBG, determinants, HbA1c, self-monitoring of blood glucose, type 2 diabetes mellitus

## Abstract

**Background and aim:**

Self-monitoring of blood glucose (SMBG) is a key component of type 2 diabetes mellitus (T2DM) management; however, adherence to recommended practices remains suboptimal. This study aimed to assess SMBG adherence among patients with T2D attending a tertiary diabetes center in Jordan and to identify factors associated with adherence.

**Methods:**

A cross-sectional study was conducted among adults with T2DM attending the National Center for Diabetes, Endocrinology, and Genetics in Amman, Jordan, between November 2022 and January 2023. Demographic and clinical data were obtained from medical records, and SMBG adherence was assessed using a validated Arabic self-reported questionnaire. Descriptive statistics summarized adherence patterns, and multivariable regression analyses were performed to identify factors independently associated with SMBG adherence.

**Results:**

Among 805 eligible participants enrolled in the study, 539 (67.0%) reported performing SMBG and were included in the adherence and practice analyses. Of these, 255 (47.3%) met the criteria for adherence to the SMBG recommendations adopted for this study. Adherence was highest among patients treated with oral hypoglycemic agents (55.3%) and lower among those receiving combined oral and insulin therapy (38.9%) or insulin alone (40.0%). SMBG was predominantly symptom-driven, and daily monitoring was uncommon. In adjusted analyses, adherence was significantly lower among patients using combined oral and insulin therapy (OR = 0.393) or insulin alone (OR = 0.425) compared with oral therapy alone. Higher adherence was independently associated with the presence of chronic kidney disease (OR = 1.789) and diabetic neuropathy (OR = 2.691). Perceived usefulness of SMBG emerged as the strongest predictor of adherence (OR = 5.741).

**Conclusion:**

Adherence to self-monitoring of blood glucose (SMBG) among patients with type 2 diabetes mellitus was suboptimal and largely symptom-driven. Adherence varied by treatment modality and the presence of diabetes-related complications and was strongly influenced by patients’ perceived usefulness of SMBG. Targeted education and individualized SMBG recommendations are needed to improve adherence in routine diabetes care.

## Introduction

Diabetes mellitus (DM) is a chronic metabolic disorder characterized by hyperglycemia resulting from defects in insulin secretion, insulin action, or both (1). Globally, the prevalence of DM, particularly type 2 diabetes mellitus (T2DM), has risen sharply. According to the International Diabetes Federation (1), approximately 589 million adults were living with diabetes in 2024, with the majority diagnosed with T2DM, and this number is projected to increase to 853 million by 2050. This rising prevalence poses a substantial public health challenge due to associated complications, healthcare costs, and the burden on healthcare systems.

In Jordan, T2DM prevalence has increased markedly over the past two decades, becoming a major public health concern. Ajlouni et al. (2) reported that DM prevalence rose from 13.0% in 1997 to 23.7% in 2017, and projections indicate that by 2050, over 1.9 million Jordanians will be living with diabetes, with 62.2% of the population affected by obesity (3). Consequently, approximately 25% of national health expenditure is expected to be directed toward managing diabetes and obesity-related conditions (3). The high prevalence of DM in Jordan is largely driven by an aging population, widespread unhealthy lifestyle behaviors, physical inactivity, and rising obesity rates (4).

Self-monitoring of blood glucose (SMBG) is a cornerstone of diabetes management, providing patients with immediate feedback to guide dietary, physical activity, and medication decisions, as well as enabling healthcare providers to assess treatment effectiveness, adjust management plans, and improve glycemic control (5–7).

In addition to SMBG, glycated hemoglobin (HbA1c) remains the principal biomarker for long-term glycemic assessment, reflecting average glucose exposure over the preceding 2 to 3 months and demonstrating a strong association with the risk of microvascular complications. While HbA1c is internationally endorsed for diagnosis and monitoring, it does not capture short-term glucose fluctuations or glycemic variability, highlighting the complementary roles of both HbA1c and SMBG in comprehensive diabetes management (8).

However, adherence to SMBG remains suboptimal and is influenced by treatment type, patient characteristics, psychological factors, and socioeconomic constraints. Older age, lower educational levels, and lower income have been associated with reduced adherence, while fear of testing, discomfort from finger pricks, and frustration with high readings further limit regular monitoring (9–12).

Socioeconomic challenges in Jordan may further limit patients’ ability to perform recommended SMBG. The cost of glucose testing supplies is substantial, with a single pack of test strips costing around US$35, requiring approximately 21 packs annually for daily monitoring, accounting for roughly 15% of an average Jordanian’s annual income (13). Insulin therapy represents an additional financial burden, contributing around 10% of annual income. These financial constraints, combined with other factors such as treatment regimen, education, and perceived complexity of self-monitoring, may contribute to low adherence to SMBG among patients with type 2 diabetes.

Moreover, optimal glycemic control extends beyond glucose monitoring alone, as lifestyle modification plays a central role in improving metabolic parameters. A randomized controlled trial demonstrated that structured weight reduction combining aerobic exercise and dietary intervention significantly improved lipid profile, insulin resistance indices, body mass index, and health-related quality of life among obese individuals with T2DM, underscoring the importance of integrated metabolic management alongside glucose monitoring (14).

In Jordan, a national study by Al-Keilani et al. (9) reported that 59% of patients were adherent to SMBG recommendations. Higher adherence was observed among patients receiving insulin therapy and those who had received structured health education regarding SMBG use. The frequency of monitoring was influenced by treatment type and testing purpose, with daily monitoring more common among patients assessing hypo- or hyperglycemia or reporting results to their physicians.

However, since that study was conducted, the prevalence of T2DM, obesity rates, healthcare costs, and economic pressures have substantially increased. Moreover, evolving treatment strategies, increased insulin utilization, and changing patient education practices may have altered adherence patterns (2, 3). Despite these shifts, recent data evaluating SMBG adherence and its determinants within the Jordanian healthcare and socioeconomic context remain limited.

While earlier studies in Jordan have reported adherence rates and selected associated factors, a comprehensive evaluation of the multidimensional patient-related, clinical, and socioeconomic determinants influencing SMBG behavior in routine practice remains insufficient. In particular, the relative contribution of treatment modality, diabetes-related complications, and patient perceptions toward SMBG adherence has not been fully elucidated within the current Jordanian context. A clearer understanding of these determinants is essential to inform targeted, context-specific interventions aimed at improving glycemic control and optimizing diabetes care delivery in Jordan.

This study was conducted to assess the level of self-monitoring of blood glucose adherence among patients with T2DM who were performing SMBG and to identify the patient-related and clinical factors associated with SMBG adherence.

## Materials and methods

### Study design and sample

A cross-sectional study was conducted among patients with T2DM attending the National Center for Diabetes, Endocrinology, and Genetics (NCDEG) in Amman, Jordan, between November 2022 and January 2023. Participants were eligible for inclusion if they were aged 18 years or older, had a confirmed diagnosis of T2DM for at least 6 months, and provided voluntary informed consent. Patients were excluded if they were pregnant or had been diagnosed with T2DM for less than 6 months or had conditions that impaired their ability to perform self-monitoring of blood glucose (SMBG), such as blindness, dementia, cognitive disability, or recent hospitalization within the past 6 months.

A systematic probability sampling technique was applied, whereby every fifth patient attending the diabetes clinics was invited to participate if they met the eligibility criteria. The minimum required sample size was determined using a standard method for estimating a population proportion with a 95% confidence level. A conservative assumption of 50% was used for the expected proportion to ensure the maximum required sample size. An acceptable margin of error of 5% was applied. Based on these assumptions, the minimum required sample size for the study was 384 participants (15).

### Data sources

Data were collected using two approaches. First, patients’ demographic and clinical information were obtained from their medical records. Second, a self-reported questionnaire was completed by each participant with the assistance of the researcher.

*Medical record data*: Information extracted from the medical records included the last three HbA1c readings, medical history, and prescribed medications. Medical history covered major comorbidities including hypertension, dyslipidemia, coronary artery disease (CAD), cerebrovascular accident (CVA), chronic kidney disease (CKD), neuropathy, and retinopathy.

*Questionnaire data*: The SMBG questionnaire (Arabic version) generated and piloted by Al-Keilani et al. (9) was used to collect data on self-monitoring adherence and practices. This tool was originally developed in English by Barnard et al. (16). The questionnaire consisted of two parts. The first section assessed patient demographics, including age, gender, height, weight, smoking status, marital status, health insurance, education, income, and comorbidities. The second section comprised 12 questions covering treatment options, duration of diabetes, the performance and frequency of self-monitoring of blood glucose (SMBG), timing and reasons for SMBG, actions taken in response to glucose readings, perceived usefulness of SMBG, and sources of information regarding the use and interpretation of SMBG results.

### Operational definitions of study variables

Weight was measured to the nearest 0.5 kg and height to the nearest 1 cm. Body mass index (BMI) was calculated as weight in kilograms divided by the square of height in meters (kg/m^2^). Participants were classified according to the World Health Organization (WHO) criteria: normal (18.5–24.9 kg/m^2^), overweight (25.0–29.9 kg/m^2^), and obese (≥30 kg/m^2^) (17).

Diabetes was diagnosed according to the American Diabetes Association (ADA) criteria, defined as an HbA1c level ≥6.5%, fasting plasma glucose ≥126 mg/dL (7.0 mmol/L), 2-h plasma glucose ≥200 mg/dL (11.1 mmol/L) during a 75-g oral glucose tolerance test, or a random plasma glucose ≥200 mg/dL (11.1 mmol/L) in the presence of classic symptoms of hyperglycemia (18). Glycated hemoglobin (HbA1c) was measured using high-performance liquid chromatography (HPLC; Bio-Rad). Controlled diabetes was defined as HbA1c ≤ 7%, and uncontrolled diabetes as HbA1c > 7% (18).

Metabolic abnormalities were defined according to the American Diabetes Association (ADA) criteria: total cholesterol ≥200 mg/dL, LDL cholesterol ≥100 mg/dL, triglycerides ≥150 mg/dL, HDL cholesterol ≤40 mg/dL in men or ≤50 mg/dL in women, or use of lipid-lowering medications (18).

Systolic and diastolic blood pressure readings were obtained using a standardized mercury sphygmomanometer, with the participant seated and the arm at heart level after at least 5 min of rest. Hypertension was defined as systolic blood pressure ≥140 mmHg, diastolic blood pressure ≥90 mmHg, or current use of antihypertensive medication (19). Renal function was classified according to KDIGO guidelines using estimated glomerular filtration rate (eGFR) and albuminuria. eGFR categories were G1 (≥90 mL/min/1.73 m^2^), G2 (60–89), G3a (45–59), G3b (20–34), G4 (15–19, 35–44), and G5 (<15 or dialysis). Albuminuria was classified by albumin-to-creatinine ratio as A1 (<30 mg/g), A2 (30–300 mg/g), and A3 (>300 mg/g) (35).

Smoking behavior was classified according to World Health Organization guidelines (36). A current smoker was defined as an individual smoking at least one cigarette per day for 1 month or longer, a former smoker was defined as someone who had smoked more than 100 cigarettes during their lifetime but had quit at the time of the study, a non-smoker was defined as a person who had never smoked or had smoked fewer than 100 cigarettes in their lifetime (36).

### Self-monitoring of blood glucose (SMBG)

Conceptually, SMBG adherence was defined as the regular performance of home blood glucose testing by individuals with T2DM. In this study, SMBG adherence was measured using an interviewer-administered questionnaire. Question 5 specifically asked: *“How often do you monitor your blood glucose levels, and how many times?”* The response options were daily, weekly, monthly, or other (including occasional monitoring when experiencing symptoms).

As there is no single universal definition of adherence to SMBG, adherence in this study was assessed according to the NCDEG Medical Committee Guidelines for the management of type 2 diabetes mellitus (T2DM). Patients on oral hypoglycemic agents were expected to perform SMBG at least once weekly, or twice weekly if occasional hypoglycemia was reported. Patients treated with both oral hypoglycemic agents and insulin were expected to perform SMBG at least once daily, while those on intensive insulin therapy (pre-mixed insulin or basal-bolus regimen) were expected to perform SMBG at least 2–3 times daily.

### Data management and statistical analysis

All data were coded and entered into SPSS version 24 (IBM Corp., Armonk, NY, USA), and data cleaning was performed using range and logical consistency checks, with any detected errors verified and corrected against the original records. Descriptive statistics were used to summarize participants’ demographic and clinical characteristics, while the overall prevalence of good SMBG adherence, as well as subgroup-specific adherence rates, was calculated. Associations between categorical variables were assessed using chi-square tests, and differences in continuous variables between groups were analyzed with independent t-tests. Multivariable binary logistic regression analyses were conducted to identify factors independently associated with SMBG adherence. All potential predictors were initially entered into the model based on clinical relevance and prior evidence. A backward elimination procedure was applied, whereby non-significant variables were sequentially removed. Only variables that remained independently and statistically significant in the adjusted model were retained in the final parsimonious model. Statistical significance was set at a two-sided *p*-value < 0.05.

### Ethical considerations

Ethical approval for this study was obtained from the NCDEG Ethical Committees (Approval No. 1/2022). Permission to use the Arabic questionnaire was obtained. All participants provided written informed consent, and data were kept confidential and used solely for research purposes.

## Results

### Participants’ characteristics

A total of 805 adults with type 2 diabetes were included, with a mean age of 60.8 years; more than half were older than 60 years. Sociodemographic and clinical characteristics of patients were shown in Table 1. Females constituted a slight majority, and over half had university or postgraduate education. One quarter of participants were current smokers. Clinically, three-quarters had diabetes for ≥5 years, and glycemic control was suboptimal in nearly two-thirds, with a mean HbA1c of 7.65%. Obesity was highly prevalent (60.6%). Most patients were treated with oral hypoglycemic agents alone, while more than one-third required combined oral therapy and insulin. Comorbidities were common, affecting almost 90% of participants, with hypertension and dyslipidemia predominating. Diabetes-related complications were frequent, particularly diabetic kidney disease, followed by retinopathy and neuropathy.

**Table 1:** Socio-demographic and clinical characteristics of patients (*n* = 805).

Variable		No	%
Age, mean (SD) = 60.81 (9.9)	20–40	23	2.9
	41–60	362	45.0
	>60	420	52.2
Gender	Male	391	48.6
	Female	414	51.4
Educational level	Primary school and below	179	22.2
	Tawjihi and secondary school	199	24.7
	University and post-graduate	427	53.0
Marital status	Single	27	3.4
	Married	655	81.4
	Widow or divorced	123	15.3
Smoking	Current smoker	201	25.0
	Ex-smoker	47	5.8
	Non-smoker	557	69.2
Monthly income (JD)	<300	208	22.2
	300–599	392	24.7
	600–1,000	177	18.6
	>1,000	28	25.7
Insurance	No	34	4.2
	Yes	771	95.8
Duration of diabetes (years)	<5	207	25.7
	≥5	598	74.3
BMI (kg/m^2^), mean (SD) = 32.19 (6.2)	Normal (18.5–24.99)	80	10.0
	Overweight (25–29.99)	236	29.4
	Obese (≥30)	487	60.6
Diabetes medications	OHA and diet	478	59.4
	OHA + and insulin	283	35.2
	Insulin	44	5.5
HBA1c, mean (SD) = 7.65 (1.6)	Controlled	307	38.2
	Uncontrolled	497	61.8
Co-morbid diseases	Yes	720	89.4
Other co-morbid diseases	Hypertension	636	88.3
	Dyslipidemia	554	76.9
	CAD	136	18.9
	CVA	45	6.3
Diabetes-related complications	Diabetic kidney disease (DKD)	392	54.4
	Neuropathy	130	18.1
	Retinopathy	259	36.0

### Self-monitoring of blood glucose

Of the 805 eligible participants enrolled, 539 reported performing SMBG and were therefore included in the adherence and practice analyses. Of the participants who performed SMBG, 255 (47.3%) were classified as adherent based on NCDEG Medical Committee recommendations. Patients with a diabetes duration of less than 5 years were significantly more likely to adhere to SMBG compared with those with a duration of 5 years or longer (57.6% vs. 44.4%, *p* = 0.011). SMBG adherence also varied significantly by treatment regimen (*p* = 0.001), with higher adherence observed among patients treated with oral hypoglycemic agents alone (55.3%) compared with those receiving combined oral hypoglycemic agents and insulin therapy (38.9%) or insulin therapy alone (40.0%).

In addition, adherence to SMBG was significantly higher among patients with diabetic neuropathy than among those without neuropathy (60.5% vs. 43.2%, *p* = 0.001). Perceived usefulness of SMBG was also strongly associated with adherence; patients who reported that SMBG was helpful demonstrated substantially higher adherence rates compared with those who did not perceive it as helpful (50.8% vs. 21.9%, *p* < 0.001).

In contrast, no significant associations were observed between SMBG adherence and age, gender, marital status, educational level, smoking status, monthly income, insurance coverage, BMI, HbA1C level, presence of other comorbidities, or ownership of a glucometer.

### SMBG-related practices and patient education

Among participants performing SMBG, important gaps in SMBG-related practices and education were identified. A total of (47.3%) were considered adherent to the NCDEG Medical Committee recommendations, based on SMBG performed using their own glucometer. Education on correct blood glucose meter use was reported by 108 participants (46.2%), while 218 participants (47.7%) reported receiving education regarding target blood glucose ranges, appropriate monitoring frequency, and recommended actions based on SMBG results.

### Frequency and timing of SMBG among patients with T2DM

The frequency of SMBG varied in this study. Twenty six percent of patients tested their blood glucose levels occasionally when having symptoms, 19.1% of them tested more than once weekly while 10.2% tested once weekly. Furthermore, 9.6, 9.1 and 8.7% tested once, twice and three times a-day, respectively, with a further 3.1% tested 4 or more times a-day. Additionally, 4.8 and 9.3% of patients tested once and twice monthly respectively, as shown in Table 2.

**Table 2:** Frequency and timing of self-monitoring of blood glucose (SMBG) among patients with T2DM who reported performing self-monitoring of blood glucose (*n* = 539).

Frequency	Total *n* (%)
Once daily	52 (9.6)
Twice daily	49 (9.1)
Three times daily	47 (8.7)
Four times daily	12 (2.2)
More than 4 times daily	5 (0.9)
Once weekly	55 (10.2)
More than once weekly	103 (19.1)
Fortnightly (Q 2 weeks)	50 (9.3)
Once monthly	26 (4.8)
Occasional periods (when having symptoms)	140 (26.0)
Timing total *n* (%)	
Before meals	305 (56.6)
2 h after meals	294 (54.6)
Before going to bed	151 (28.0)
When waking up	334 (62.0)
During episode(s) of illness	321 (59.6)
If I feel that I am having a hypoglycemic episode	399 (74.2)
If I feel my blood glucose level is higher than it should be	370 (69.0)
before/during/after any physical activity	21 (3.9)
A change in my treatment is being considered	157 (29.1)
To monitor the effect of certain foods	217 (40.4)

The timing of self-monitoring blood glucose (SMBG) was mainly when the patients feel hypo-hyperglycemic (74.2 and 69%) respectively. With 62, 59.6, 56.6 and 54.6% of patients performed SMBG when waking up, during episodes of illness, before meals and 2-h after meals, respectively. Moreover, 40.4 and 29.1% of participants performed SMBG to monitor the effect of certain foods or when a change in treatment was considered, respectively. Self-Monitoring of Blood Glucose was performed just before going to bed in 28%.

### Reasons for SMBG

Reasons for self-monitoring of blood glucose (SMBG) varied among participants, as shown in Table 3. Participants reporting the highest reason for SMBG was to confirm if there was a hypoglycemic episode in (*n* = 405, 75.1%) or a high blood glucose level in (*n* = 397, 74.3%), followed by the reason for knowing the effect of diabetes on body organs in (*n* = 372, 69%), and to make adjustment to their food intake in (*n* = 288, 53.6%). However, the lowest reported reason was to monitor blood glucose levels in case of being a pregnant woman (*n* = 3, 0.6%) followed by when assessing the impact of physical activity (*n* = 21, 4%).

**Table 3:** Reasons for self-monitoring of blood glucose and actions taken in response to blood glucose readings among patients with T2DM who reported performing self-monitoring of blood glucose (*n* = 539).

Reason	Total *n* (%)
To make adjustments to my food intake	288 (53.6)
When assessing the impact of physical activity	21 (3.9)
To confirm if I’m having a hypoglycemic episode	405 (75.1)
To confirm if I have a high blood glucose level	397 (74.3)
For knowing effect of diabetes on body organs such as (eyes, neurons, kidneys, etc.)	372 (69.0)
To inform me about the effect of a new diabetes medication I’ve started	113 (21.0)
To inform me about my blood glucose levels during a period of illness	242 (45.0)
To monitor my blood glucose levels as I’m plan to be pregnant	3 (0.6)
To inform my doctor	253 (47.0)
Action taken in response	Total *n* (%)
I adjusted my medication	167 (31.3)
I did more physical activity	110 (20.6)
I adjusted my carbohydrate for the same mealtime the next day	327 (61.1)
I contacted a member of my diabetes healthcare team for advice (Doctor, Nurse)	58(11.0)
I wait my next doctor visit to inform him	252 (47.1)
I did not do anything	99 (18.5)

More than one choice was allowed.

### Actions taken by participants in response to an unexpected blood glucose reading

In response to unexpected blood glucose readings, the most common actions reported by participants were adjusting their carbohydrate intake for the same mealtime the next day (*n* = 327, 61%) followed by waiting for their next doctor’s visit to inform them (*n* = 252, 47.1%). Additionally, contacting a member of their diabetes healthcare team, such as a doctor or nurse, for immediate advice was the least common response, with only 11% of participants reporting this action, (*n* = 58, 11%), as shown in Table 3.

### Factors associated with adherence to self-monitoring of blood glucose

Multiple logistic regression analysis was performed to identify factors independently associated with adherence to self-monitoring of blood glucose (SMBG). Compared with participants treated with oral hypoglycemic agents (OHA) alone, adherence to SMBG was significantly lower among those receiving combined OHA and insulin therapy (OR = 0.393; 95% CI: 0.183–0.843) and among those treated with insulin alone (OR = 0.425; 95% CI: 0.288–0.628).

Participants with chronic kidney disease were significantly more likely to adhere to SMBG compared with those without chronic kidney disease (OR = 1.789; 95% CI: 1.181–2.710). Similarly, participants with diabetic neuropathy demonstrated higher adherence to SMBG than those without neuropathy (OR = 2.691; 95% CI: 1.732–4.180).

In addition, participants who reported high satisfaction with the helpfulness and usefulness of SMBG were substantially more likely to adhere to SMBG compared with those who perceived SMBG as not helpful (OR = 5.741; 95% CI: 2.936–11.224). These findings are summarized in Table 4.

**Table 4:** Factors associated with adherence to self-monitoring of blood glucose.

Variable	OR	95% confidence interval	*p*-value
Diabetes medications			
OHA	1		
OHA + insulin	0.393	0.183–0.843	0.016
Insulin	0.425	0.288–0.628	<0.001
Neuropathy			
No	1		<0.001
Yes	2.691	1.732–4.180	
CKD			
No	1		0.006
Yes	1.789	1.181–2.710	
How helpful or unhelpful do you find self-monitoring blood glucose (SMBG)?			
Not helpful	1		<0.001
Helpful	5.741	2.936–11.224	

## Discussion

Adherence to self-monitoring of blood glucose (SMBG) varies widely across populations, treatment regimens, and healthcare settings. In the present study, conducted in accordance with the NCDEG Medical Committee Guidelines, 47.3% of participants with type 2 diabetes mellitus (T2DM) adhered to recommended SMBG practices, indicating a moderate level of adherence. Comparable adherence rates have been reported in similar contexts. In Ethiopia, Endale et al. (37) found that 43.7% of adults with T2DM demonstrated good adherence to diabetes self-care practices, including SMBG. Likewise, a U.S. retrospective cohort study by Whitley et al. (38) reported 49% adherence to SMBG, closely aligning with our findings; notably, SMBG-adherent patients in that study were less likely to experience diabetes-related hospitalization, suggesting potential benefits of SMBG beyond glycemic control.

Marked regional variation in SMBG adherence has been reported across different healthcare settings. Higher adherence rates have been documented in some regions; for example, a systematic review and meta-analysis by Paudel et al. (39) reported relatively high SMBG adherence in South Asian countries, including 68% in India and 66% in Pakistan. In the Middle East, Al-Keilani et al. (9) reported a moderate adherence rate of 59% in a multicenter study across 18 hospitals and healthcare centers in Jordan. Consistent with these findings, a cross-sectional study from Saudi Arabia by AlRasheed et al. (40) found moderate SMBG adherence in 58.5% of adults with T2DM, with only a minority achieving high adherence. In contrast, studies from low- and middle-income countries have reported low adherence to self-monitoring of blood glucose (SMBG). For example, a study conducted at Debre Tabor Comprehensive Specialized Hospital in Ethiopia found that only 37.2% of patients practiced home blood glucose monitoring using a glucometer (41).

Collectively, these findings indicate that adherence to SMBG among individuals with T2DM remains suboptimal worldwide, with substantial heterogeneity across regions. Such variability likely reflects differences in treatment regimens, the absence of standardized SMBG frequency guidelines, and a range of sociodemographic and health system–related factors, including limited access to healthcare services and diabetes care resources, the affordability and availability of glucose monitoring supplies, variability in diabetes education, and differences in clinical recommendations and patient perceptions regarding the usefulness of SMBG.

In the present study, SMBG adherence was highest among patients treated with oral hypoglycemic agents (55.3%), compared with those receiving combined oral and insulin therapy or insulin alone, indicating that treatment modality may meaningfully influence monitoring behavior. This pattern is supported by evidence from China. Wang et al. (42) reported low overall SMBG adherence (27.5%) among 721 patients with T2DM; however, adherence varied by regimen and was highest among patients using oral agents (36.6%). In that study, oral therapy, diet and exercise-based management, glucometer ownership, and higher educational level were independently associated with better adherence. Consistent with these findings, Yuan et al. (12) observed very low overall adherence (19%) in a large Chinese cohort (*n* = 5,953), with substantially poorer adherence among insulin users compared with those treated with oral agents (7% vs. 39%), further reinforcing the influence of treatment regimen on SMBG practice. However, findings across studies are not entirely consistent. Hu et al. (43) reported higher overall adherence (57.6%) using 90-day real-world monitoring data yet found that longer diabetes duration and oral antidiabetic use were independently associated with poorer adherence. These results suggest that the relationship between medication type and SMBG behavior may be modified by disease duration, patient perceptions, and treatment-related burden.

In the present study, substantial variability was observed in both the frequency and timing of self-monitoring of blood glucose (SMBG) among individuals with type 2 diabetes mellitus (T2DM). Approximately one quarter of participants (26%) reported testing their blood glucose only occasionally when experiencing symptoms, while 19.1% tested more than once weekly and 10.2% tested once weekly. Daily monitoring was uncommon: 9.6, 9.1, and 8.7% of participants reported testing once, twice, or three times per day, respectively, and only 3.1% reported testing four or more times daily. These findings indicate that regular, structured SMBG remains infrequent in this population.

Overall, most participants did not adhere to structured or guideline-based SMBG schedules, irrespective of treatment regimen. Instead, monitoring behavior was largely symptom-driven, with the majority reporting glucose testing primarily in response to symptoms suggestive of hypoglycemia (74.2%) or hyperglycemia (69%). This reactive pattern suggests that SMBG is often perceived as a tool for confirming acute glycemic disturbances rather than as an integral component of routine diabetes self-management. In the current study, the primary motivation for performing SMBG was to verify suspected episodes of hypo- or hyperglycemia. Following glucose testing, most participants reported either modifying carbohydrate intake in an unsystematic manner or deferring interpretation of results until their next clinical encounter, rather than making informed, real-time self-management decisions. This behavior indicates limited confidence in autonomous diabetes self-management and likely reflects gaps in structured diabetes education, particularly with respect to interpreting SMBG results and translating them into appropriate therapeutic actions.

Similar motivations and response patterns have been reported in previous regional studies. Al-Keilani et al. (9) whose study was also conducted in Jordan, reported that most patients performed SMBG only when they perceived symptoms of hypoglycemia (48.9%) or hyperglycemia (48.0%). Comparable patterns have been documented in neighboring settings; for example, a study from Iraq by Salih et al. (44) reported that 62% of patients monitored their blood glucose exclusively during symptomatic episodes. Together, these findings suggest that symptom-triggered SMBG remains a dominant practice in Middle Eastern contexts.

More recent international evidence further confirms that adherence to recommended self-monitoring of blood glucose (SMBG) frequency remains suboptimal across diverse settings. In a large cross-sectional study conducted in China, Lin et al. (20) reported that only 26.2% of 1,388 adults with type 2 diabetes mellitus met standard SMBG compliance criteria. Similarly, a 2024 cross-sectional study from Bangladesh found that only half of participants (50.4%) used a glucometer, with lack of awareness cited as the main barrier to use; among users, glucose monitoring was most commonly performed weekly (21). Collectively, these findings highlight a persistent gap between guideline-based SMBG recommendations and real-world practice, particularly in low- and middle-income settings.

Taken together, the available evidence indicates that among Jordanian patients with T2DM, SMBG practices remain predominantly episodic, and symptom triggered. Limited diabetes education, low self-efficacy in interpreting glucose results, and the financial burden associated with testing supplies likely contribute to inconsistent and non-purposeful monitoring behaviors. In contrast, evidence from higher-resource healthcare contexts suggests that a greater proportion of patients engage in more routine SMBG, with regular daily monitoring reported in a meaningful subset of patients (22, 23). Moreover, recent analyses from high-income settings have linked more frequent and structured SMBG use with modest improvements in glycemic outcomes, underscoring the potential benefits of routine monitoring when supported by comprehensive education and healthcare infrastructure (22, 23).

In this context, long-term glycemic status is traditionally assessed using glycated hemoglobin (HbA1c), which reflects cumulative glycemic exposure over approximately 3 months and is strongly associated with the development of microvascular complications. However, HbA1c represents an averaged measure and does not capture daily glucose excursions, hypoglycemic episodes, or glycemic variability (8). In addition, its accuracy may be affected by clinical conditions that alter erythrocyte lifespan or hemoglobin structure, including hemolysis, anemia, hemoglobinopathies, renal impairment, and advancing age, potentially resulting in discrepancies between measured HbA1c values and true glycemic exposure. Current evidence therefore supports a complementary approach in which SMBG provides real-time, actionable data, while HbA1c offers longitudinal risk stratification, together enabling a more comprehensive and individualized evaluation of glycemic control in individuals with T2DM.

Improving SMBG practices in this setting requires multifaceted interventions. Strategies should prioritize enhancing access to affordable glucose monitoring supplies, integrating structured diabetes education into routine clinical care, and strengthening patient empowerment through behavioral support and self-management training. Such targeted efforts have the potential to transform SMBG from a reactive behavior into an effective tool for ongoing glycemic management, which may ultimately improve glycemic control and reduce diabetes-related complications among individuals with T2DM. Furthermore, comprehensive diabetes care extends beyond glucose monitoring alone. Evidence from interventional research demonstrates that structured lifestyle modification, particularly programs combining aerobic exercise and caloric restriction, can significantly improve body mass index, lipid profile parameters, and insulin resistance indices, while simultaneously enhancing health-related quality of life in individuals with T2DM (14). These findings underscore that effective glycemic management requires an integrated approach in which behavioral interventions and metabolic optimization complement glucose monitoring practices.

The presence of chronic kidney disease (CKD) was independently associated with higher adherence to self-monitoring of blood glucose (SMBG) among patients with type 2 diabetes mellitus (T2DM). This association may reflect the greater clinical complexity and perceived disease severity associated with advanced diabetes and its complications, particularly in patients at increased risk of hypoglycemia and other adverse outcomes, thereby necessitating closer glucose surveillance. Previous evidence indicates that diabetes self-care behaviors are strongly influenced by disease burden, perceived risk, and the presence of complications. In a mixed-methods study from Northern Ethiopia, Zewdie et al. (24) reported that although only half of patients with T2DM demonstrated adequate self-care practices, individuals with diabetes-related complications exhibited significantly better adherence, suggesting that complications may act as a catalyst for enhanced self-management behaviors. Similarly, Portela et al. (25) reported higher adherence to SMBG among patients with diabetes-related complications in Brazil. A hospital-based study from Eastern Ethiopia by Ayele et al. (26) further demonstrated that a high perceived severity of diabetes and its complications was a strong predictor of self-care behaviors, increasing the likelihood of self-care by more than twelvefold. These findings are further supported by van Puffelen et al. (27) who showed that among recently diagnosed patients, the presence of complications was associated with perceiving diabetes as more serious and less predictable, which in turn was linked to higher engagement in specific self-care behaviors such as exercise and foot care.

Collectively, this body of evidence supports the notion that the presence of chronic kidney disease (CKD) may heighten risk awareness and increase clinical engagement, thereby reinforcing adherence to self-monitoring of blood glucose (SMBG) and other diabetes self-management behaviors. Consistent with this interpretation, Yeoh et al. (28) demonstrated that SMBG was associated with improved short-term glycemic control among patients with type 2 diabetes mellitus (T2DM) and diabetic kidney disease, without an increased risk of hypoglycemia, underscoring the clinical value of intensified glucose monitoring in this high-risk population. The observed association between CKD and SMBG adherence in the present study may therefore reflect the need for stricter glycemic monitoring in the context of limited therapeutic flexibility and an elevated risk of hypoglycemia. Nevertheless, conflicting evidence exists; Lim et al. (29) reported low SMBG scores among patients with diabetic CKD, potentially attributable to barriers such as finger-prick discomfort, the cost of testing supplies, and a greater focus on medication adherence and dietary management rather than routine glucose monitoring.

Notably, this study found that patients with diabetic neuropathy were more likely to adhere to self-monitoring of blood glucose (SMBG). A plausible explanation is that the presence of neuropathy may increase patients’ awareness of disease progression and vulnerability to further complications, thereby motivating greater engagement in glucose monitoring. Neuropathic symptoms, including pain and sensory disturbances, may also prompt more frequent healthcare interactions and reinforce the importance of maintaining close glycemic control. This interpretation aligns with prior research suggesting that diabetes-related complications can function as behavioral triggers for improved self-care practices. Although some studies have linked diabetic neuropathy to poorer self-care behaviors and greater psychological distress, including depressive symptoms, the present findings suggest that, in certain contexts, neuropathy may heighten perceived risk and encourage adherence to SMBG (30, 31). These observations highlight the complex and potentially bidirectional relationship between diabetes complications and self-management behaviors, underscoring the importance of individualized patient education and support.

In the present study, patients who perceived self-monitoring of blood glucose (SMBG) as helpful demonstrated significantly higher adherence, indicating that patients’ belief in the usefulness of SMBG is a key determinant of adherence. This finding is consistent with prior evidence emphasizing the role of internal psychological and cognitive factors in SMBG engagement. Mak and Lau (32) reported that greater health consciousness, higher perceived self-efficacy, stronger motivation, and a clearer understanding of the usefulness of SMBG were associated with improved adherence, with educational attainment and diabetes-related knowledge further reinforcing monitoring practices. Complementary patient-centered evidence is provided by Pfoh et al. (33) who reported that among non–insulin-treated individuals with type 2 diabetes, SMBG was often valued for its psychological and informational benefits, including reduced worry, improved quality of life, and better understanding of disease status. Importantly, patients who monitored blood glucose for intrinsic reasons, such as habit or personal insight, were more likely to continue SMBG than those who monitored primarily in response to external instruction (33). From a provider perspective, Hamshari et al. (34) found that primary healthcare physicians generally held positive attitudes toward SMBG and recognized its importance for glycemic control and prevention of microvascular complications. However, substantial gaps in confidence related to SMBG data interpretation and treatment adjustment were identified, and fewer than half of physicians routinely recommended frequent SMBG to newly diagnosed patients. Taken together, these findings suggest that although both patients and clinicians acknowledge the value of SMBG, limitations in confidence, education, and practical implementation may hinder the translation of perceived usefulness into consistent, SMBG-supported self-management.

In the present study, demographic and clinical characteristics, including age, gender, marital status, educational level, smoking status, monthly income, insurance, BMI, HbA1c, and possession of a glucometer, were not associated with adherence to self-monitoring of blood glucose (SMBG). These findings are consistent with Al-Keilani et al. (9) who similarly reported no significant association between SMBG frequency and these demographic variables. Conversely, a Brazilian cross-sectional study examining sociodemographic factors related to adherence to self-care activities in T2DM identified associations between BMI and educational attainment and SMBG adherence, with underweight individuals and those with higher educational levels demonstrating greater adherence to glycemic testing (25).

This study has several strengths and limitations. It provides timely, locally relevant evidence on SMBG adherence in Jordan, utilizing a large, systematically sampled cohort from a national tertiary center and validated measurement tools. Nonetheless, its single-center, cross-sectional design may limit generalizability and does not allow conclusions about cause-and-effect relationships. Reliance on self-reported data introduces potential recall bias. Furthermore, important psychosocial and environmental factors, such as family support, financial challenges, and emotional well-being, were not assessed and should be explored in future studies.

## Conclusion

This study demonstrates that adherence to self-monitoring of blood glucose (SMBG) among individuals with type 2 diabetes mellitus (T2DM) remains suboptimal, despite being conducted in accordance with the NCDEG Medical Committee Guidelines. Fewer than half of participants (47.3%) adhered to recommended SMBG practices. Adherence was highest among patients treated with oral hypoglycemic agents and substantially lower among those receiving insulin alone or combined insulin and oral therapy, underscoring the important influence of treatment modality on monitoring behavior.

SMBG practices in this population were predominantly episodic and symptom-driven, with most patients testing their blood glucose primarily in response to symptoms of hypoglycemia or hyperglycemia rather than as part of a structured self-management routine. Following glucose testing, many participants reported modifying carbohydrate intake in an unsystematic manner or deferring interpretation of results until their next clinical encounter, rather than making informed, real-time self-management decisions. These patterns reflect limited confidence in autonomous diabetes management and highlight persistent gaps in diabetes self-management education.

Clinical factors were also associated with adherence to self-monitoring of blood glucose (SMBG). Higher adherence was observed among patients with comorbid chronic kidney disease, which may reflect greater perceived disease severity and closer clinical monitoring. In addition, patients with diabetic neuropathy demonstrated higher adherence to SMBG, potentially due to increased awareness of disease progression and perceived vulnerability to further complications. Moreover, patients who perceived SMBG as helpful exhibited significantly higher adherence, underscoring the importance of patient beliefs and perceived benefit in influencing self-management behaviors. Taken together, these findings indicate that SMBG adherence is shaped by treatment complexity, comorbidity burden, and patient education, and remains insufficiently integrated into routine diabetes self-management for many individuals with T2DM.

## Recommendations

Based on these findings, multifaceted and context-specific interventions are required to improve SMBG adherence and effectiveness among individuals with T2DM. Structured diabetes education programs should be strengthened, with particular emphasis on improving patients’ ability to interpret SMBG results and translate them into appropriate, real-time self-management actions. Education efforts should move beyond symptom recognition and focus on the preventive and regulatory role of routine SMBG in achieving glycemic control.

Healthcare providers should adopt individualized SMBG recommendations that account for treatment modality, comorbidity profile, and patient capacity, with particular attention to patients receiving insulin therapy and those with diabetic neuropathy, who demonstrated lower adherence. Integrating SMBG counseling into routine clinical visits, reinforcing patient self-efficacy, and providing practical guidance on dietary and therapeutic adjustments may enhance the meaningful use of SMBG data.

At the system level, improving access to affordable glucose monitoring supplies and reducing the financial burden of testing are essential to support sustained SMBG practice. Additionally, standardized SMBG education aligned with national guidelines should be incorporated into primary care services to promote consistent messaging and practice. Together, these strategies have the potential to transform SMBG from a reactive, symptom-driven behavior into an effective component of ongoing diabetes self-management, ultimately supporting improved glycemic control and reducing diabetes-related complications among individuals with T2DM.

## Data Availability

The raw data supporting the conclusions of this article will be made available by the authors, without undue reservation.

## References

[1] International Diabetes Federation. IDF Diabetes Atlas. 11th Edn. Brussels: International Diabetes Federation (2025). Available online at: https://www.idf.org/our-activities/advocacy-awareness/resources-and-tools/133:idf-diabetes-atlas.html (Accessed March 12, 2026).

[2] Ajlouni K Batieha A Jaddou H Khader Y Abdo N El-Khateeb M . Time trends in diabetes mellitus in Jordan between 1994 and 2017. Diabet Med. (2019) 36:1176–82. doi: 10.1111/dme.13894, 30614070

[3] Awad SF Huangfu P Dargham SR Ajlouni K Batieha A Khader YS . Characterizing the type 2 diabetes mellitus epidemic in Jordan up to 2050. Sci Rep. (2020) 10:21001. doi: 10.1038/s41598-020-77970-7, 33273500 PMC7713435

[4] Centers for Disease Control and Prevention. National Diabetes Statistics Report. Atlanta, GA: CDC (2024). Available online at: https://www.cdc.gov/diabetes/php/data-research/index.html (Accessed March 12, 2026).

[5] Blevins T. Value and utility of self-monitoring of blood glucose in non-insulin-treated patients with type 2 diabetes mellitus. Postgrad Med. (2013) 125:191–204. doi: 10.3810/pgm.2013.05.2668, 23748520

[6] William AF Cornman DH Kohut T Schachner H Stenger P. What primary care providers can do to address barriers to self-monitoring of blood glucose. Clin Diabetes. (2013) 31:34–42. doi: 10.2337/diaclin.31.1.34

[7] Miller KM Beck RW Bergenstal RM Goland RS Haller MJ McGill JB . Evidence of a strong association between frequency of self-monitoring of blood glucose and hemoglobin A1c levels in T1D exchange clinic registry participants. Diabetes Care. (2013) 36:2009–14. doi: 10.2337/dc12-1770, 23378621 PMC3687326

[8] Tashkandi M. Revisiting HbA1c in type 2 diabetes: strengths, pitfalls, and emerging alternatives. Glob J Basic Sci. (2025) 1:1–8. doi: 10.63454/jbs20000055

[9] Al-Keilani MS Almomani BA Al-Sawalha NA Shhabat BA. Self-monitoring of blood glucose among patients with diabetes in Jordan: perception, adherence, and influential factors. Diabetes Res Clin Pract. (2017) 126:79–85. doi: 10.1016/j.diabres.2017.01.005., 28236721

[10] Ong WM Chua SS Ng CJ. Barriers and facilitators to self-monitoring of blood glucose in people with type 2 diabetes using insulin: a qualitative study. Patient Prefer Adherence. (2014) 8:237–46. doi: 10.2147/PPA.S57567, 24627628 PMC3931581

[11] Karter AJ Ferrara A Darbinian JA Ackerson LM Selby JV. Self-monitoring of blood glucose: language and financial barriers in a managed care population with diabetes. Diabetes Care. (2000) 23:477–83. doi: 10.2337/diacare.23.4.477, 10857938

[12] Yuan L Guo X Xiong Z Lou Q Shen L Zhao F . Self-monitoring of blood glucose in type 2 diabetic patients in China: current status and influential factors. Chin Med J. (2014) 127:201–7. doi: 10.3760/cma.j.issn.0366-6999.20131776, 24438604

[13] Department of Statistics – Jordan (2025) Jordan Statistical Yearbook 2024 Amman, Jordan Department of Statistics. Available online at: https://dosweb.dos.gov.jo/product/jordan-statistical-yearbook-2024/ (Accessed March 12, 2026).

[14] Qahwaji RQ Sharma PK Mobashir M. Biochemical parameters and quality of life response to lifestyle modification in obese with type 2 diabetes mellitus. Glob J Basic Sci. (2025) 1:1–8. doi: 10.63454/jbs20000023.

[15] Cochran WG. Sampling Techniques. 3rd ed. New York: Wiley (1977).

[16] Barnard KD Young AJ Waugh NR. Self monitoring of blood glucose - a survey of diabetes UK members with type 2 diabetes who use SMBG. BMC Res Notes. (2010) 3:318. doi: 10.1186/1756-0500-3-318, 21092171 PMC2998520

[17] World Health Organization. Obesity and Overweight: Definition and Facts Geneva: World Health Organization (2025). Available online at: https://www.who.int/news-room/fact-sheets/detail/obesity-and-overweight (Accessed March 12, 2026).

[18] American Diabetes Association Professional Practice Committee. 2. Diagnosis and classification of diabetes: standards of care in diabetes-2024. Diabetes Care. (2024) 47:S20–S42. doi: 10.2337/dc24-S002, 38078589 PMC10725812

[19] American Diabetes Association Professional Practice Committee. 10. Cardiovascular disease and risk management: standards of care in diabetes–2024. Diabetes Care. (2024) 47:S179–218. doi: 10.2337/dc24-S010, 38078592 PMC10725811

[20] Lin M Chen T Fan G. Current status and influential factors associated with adherence to self-monitoring of blood glucose with type 2 diabetes mellitus patients in grassroots communities: a cross-sectional survey based on information-motivation-behavior skills model in China. Front Endocrinol (Lausanne). (2023) 14:1111565. doi: 10.3389/fendo.2023.1111565, 37441499 PMC10335788

[21] Kabir MA Ahmed S Abedin M Mallick S Dhar A Islam AS . Prevalence and predictors of home use blood glucose meters among the people with diabetes in Bangladesh and relation with HbA1c: a multicenter cross-sectional study. Glob J Diabetes Endocrinol Metab Disord. (2022) 1:05–22. doi: 10.52402/Diabetes702

[22] Muhali SS Muhali FS Mfinanga SG Sadiq AM Marandu AA Kyala NJ . Impact of self-monitoring blood glucose on glycaemic control among insulin-treated patients with diabetes mellitus in northeastern Tanzania: a randomised controlled trial. J Diabetes Res. (2024) 2024:6789672. doi: 10.1155/2024/6789672, 38899147 PMC11186681

[23] Holmes-Truscott E Baptista S Ling M Collins E Ekinci EI Furler J . The impact of structured self-monitoring of blood glucose on clinical, behavioral, and psychosocial outcomes among adults with non-insulin-treated type 2 diabetes: a systematic review and meta-analysis. Front Clin Diabetes Healthcare. (2023) 4:1177030. doi: 10.3389/fcdhc.2023.1177030, 37153750 PMC10157033

[24] Zewdie S Moges G Andargie A Habte BM. Self-care practice and associated factors among patients with type 2 diabetes mellitus at a referral Hospital in Northern Ethiopia - a mixed methods study. Diabetes Metab Syndr Obes. (2022) 15:3081–91. doi: 10.2147/DMSO.S373449, 36237969 PMC9553239

[25] Portela RA Silva JRS Nunes FBBF Lopes MLH Batista RFL Silva ACO. Diabetes mellitus type 2: factors related to adherence to self-care. Rev Bras Enferm. (2022) 75:e20210260. doi: 10.1590/0034-7167-2021-0260, 35239839

[26] Ayele K Tesfa B Abebe L Tilahun T Girma E. Self care behavior among patients with diabetes in Harari, eastern Ethiopia: the health belief model perspective. PLoS One. (2012) 7:e35515. doi: 10.1371/journal.pone.0035515, 22530039 PMC3328462

[27] van Puffelen AL Heijmans MJ Rijken M Rutten GE Nijpels G Schellevis FG . Illness perceptions and self-care behaviours in the first years of living with type 2 diabetes; does the presence of complications matter? Psychol Health. (2015) 30:1274–87. doi: 10.1080/08870446.2015.1045511, 25925788

[28] Yeoh E Lim BK Fun S Tong J Yeoh LY Sum CF . Efficacy of self-monitoring of blood glucose versus retrospective continuous glucose monitoring in improving glycaemic control in diabetic kidney disease patients. Nephrology (Carlton). (2018) 23:264–8. doi: 10.1111/nep.12978, 27933715

[29] Lim GJ Low S Liu AYL Shao YM Subramaniam T Sum CF . Association between self-care and chronic kidney disease in patients with type 2 diabetes mellitus. Ann Acad Med Singap. (2023) 52:52–4. doi: 10.47102/annals-acadmedsg.2022299, 36730807

[30] Timar B Timar R Schiller A Oancea C Roman D Vlad M . Impact of neuropathy on the adherence to diabetes-related self-care activities: a cross-sectional study. Patient Prefer Adherence. (2016) 10:1169–75. doi: 10.2147/PPA.S107621, 27445464 PMC4936822

[31] Rekleiti M Sarafis P Saridi M Toska A Melos C Souliotis K . Investigation of depression in Greek patients with diabetic peripheral neuropathy. Glob J Health Sci. (2013) 5:107–14. doi: 10.5539/gjhs.v5n5p107, 23985112 PMC4776877

[32] Mak WH Lau RW. Predictors of self-monitoring of blood glucose among noninsulin-treated patients with type 2 diabetes in a primary care setting in Hong Kong: a cross-sectional study. SAGE Open Med. (2021) 9:20503121211066150. doi: 10.1177/20503121211066150, 34992780 PMC8724976

[33] Pfoh ER Linfield D Speaker SL Roufael JS Yan C Misra-Hebert AD . Patient perspectives on self-monitoring of blood glucose when not using insulin: a cross-sectional survey. J Gen Intern Med. (2022) 37:1673–9. doi: 10.1007/s11606-021-07047-2, 34389935 PMC9130377

[34] Hamshari S Hamadneh S Ajlone AM Mashni AW Jubeh MA. Knowledge, attitude, and practice (KAP) of primary health physicians towards glucose self-monitoring in patients with type2 diabetes mellitus in Palestine. BMC Prim Care. (2025) 26:33. doi: 10.1186/s12875-025-02720-5, 39930336 PMC11809109

[35] Kidney Disease. Improving global outcomes (KDIGO) CKD work group. KDIGO 2012 clinical practice guideline for the evaluation and Management of Chronic Kidney Disease. Kidney Int Suppl. (2013) 3:1–150.

[36] World Health Organization. Guidelines for Controlling and Monitoring the Tobacco Epidemic. Geneva: World Health Organization (1998).

[37] Endale A Hundessa F Tamru E Nigussie F Hailu M. Adherence to diabetic self-care management and associated factors among type 2 diabetic patients in north Shewa zone public hospitals in Amhara region, Ethiopia. Front Clin Diabetes Healthcare. (2025) 6:1560907. doi: 10.3389/fcdhc.2025.1560907, 40416402 PMC12098080

[38] Whitley MY Van Wieren Jones EM Hansen BK Vora J. The impact of self-monitoring blood glucose adherence on glycemic goal attainment in an indigent population, with pharmacy assistance. PT. (2019) 44:554–9. PMC6705474 31485151

[39] Paudel G Vandelanotte C Dahal PK Biswas T Yadav UN Sugishita T . Self-care behaviours among people with type 2 diabetes mellitus in South Asia: a systematic review and meta-analysis. J Glob Health. (2022) 12:04056. doi: 10.7189/jogh.12.04056, 35916498 PMC9346342

[40] AlRasheed AY Hashim H Alrofaie H. Adherence to self-monitoring of blood glucose and its related factors among type 2 diabetic patients attending Al-Ahsa primary health care centers in Saudi Arabia. Cureus. (2024) 16:e65545. doi: 10.7759/cureus.65545, 39188431 PMC11346824

[41] Ewunetu M Belay BM Abere Y Melkamu B Eshetie Y Dires T. Home self monitoring of blood glucose using a glucometer and its determinants among diabetes mellitus. Sci Rep. (2025) 15:17217. doi: 10.1038/s41598-025-02196-4, 40382440 PMC12085629

[42] Wang X Luo JF Qi L Long Q Guo J Wang HH. Adherence to self-monitoring of blood glucose in Chinese patients with type 2 diabetes: current status and influential factors based on electronic questionnaires. Patient Prefer Adherence. (2019) 13:1269–82. doi: 10.2147/PPA.S211668, 31413552 PMC6662864

[43] Hu ZD Zhang KP Huang Y Zhu S. Compliance to self-monitoring of blood glucose among patients with type 2 diabetes mellitus and its influential factors: a real-world cross-sectional study based on the Tencent TDF-I blood glucose monitoring platform. Mhealth. (2017) 3:25. doi: 10.21037/mhealth.2017.06.01, 28736734 PMC5505930

[44] Salih AA Sadiq MA Rayed MH. Self-monitoring of blood glucose, practices, and determinants in type 2 diabetics. Prev Med Commun Health. (2021) 4:157. doi: 10.15761/PMCH.1000157

