# Correction: Determinants of self-monitoring adherence in type 2 diabetes: bridging the gap in patient care

**DOI:** 10.3389/fpubh.2026.1859246

**Published:** 2026-05-07

**Authors:** Dana Hyassat, Kawther Alonezat, Maysoon Abdalrahim, Anwar Batieha, Yousef Khader, Sara Alnsour, Sarah B. Al-Qalalweh, Kamel Ajlouni

**Affiliations:** 1 National Center for Diabetes, Endocrinology and Genetics, The University of Jordan, Amman, Jordan; 2 School of Nursing, The University of Jordan, Amman, Jordan; 3 Department of Epidemiology and Biostatistics, Jordan University of Science and Technology, Irbid, Jordan; 4 School of Medicine, The University of Jordan, Amman, Jordan

**Keywords:** adherence to SMBG, determinants, HbA1c, self-monitoring of blood glucose, type 2 diabetes mellitus

Author Kawther Alonezat was erroneously assigned as equal contributing author. The original version of this article has been updated.

